# Evolution of Tubercle

**Published:** 1842-03

**Authors:** 


*Evolution of Tubercle.*—Dr. C. Haller has had ample opportunity of observing the various forms in which tubercles in the lungs are evolved, in the House of Correction, Vienna, where phthisis is extremely common, "where *lung-tuberculosis* is endemic," as the author says. Here, as everywhere else, the most common of all the forms of tubercular development in the lungs, is, 1st, that of a *bronchial catarrh*, or seemingly simple cough, with or without febrile symptoms, and accompanied either with very little or no expectoration; 2d, often the disease commenced in the guise of *pneumonia;* 3d, still more frequently it assumed the appearance of a common continued fever; and, 4th, in rare instances it presented itself with the perfect tertian or quotidian intermittent type.

The first form is sufficiently well known—description has been exhausted in regard to it. But the form in which pneumonia is simulated, has attracted less attention. Here violent fever, with great dyspnœa, or rather breathlessness, a sensation as if a load lay upon the breast, without much or any positive pain referred to a particular spot, distressing and almost dry cough attacking in fits, violent action of the heart, with an accelerated, hard, and often irregular pulse, a burning skin, great sense of depression, and rapid sinking—all the symptoms, in short, indicating acute pneumonia; but this diagnosis is not borne out by the information conveyed by auscultation and percussion. The chest sounds well everywhere; it is even tympanitic in its tone. The respiratory murmur is appreciable at every part, only somewhat rough, with occasional small, but, by degrees, increasing, mucous rattle; in a word, auscultation and percussion lead only to negative results.

The symptoms continue for several days unabated either by blood-letting or the treatment generally followed under the same circumstances, and, so far as symptoms are concerned, with success; though, if the exudation of tubercular matter be extensive, the patient may be lost within from a week to a fortnight. If the tubercular exudation have been limited to the upper part of the lungs, the symptoms are less severe, and there is less immediate and apparent danger; the dyspnœa becomes less by degrees, the cough becomes moister, but the febrile state continues long, and, when it ends, it is without a crisis of any kind. The little relief procured by blood-letting, the great amount of dyspnœa, the slight indications of inflammation presented by the blood that is abstracted, the negative results of auscultation, the pertinacity of the fever, and the want of critical phenomena, particularly by urine, suffice to distinguish this disease from true pneumonia.

From proper typhus fever the diagnosis of tubercular formation is more difficult. But the disease in this case is always preceded for a long time by a greater or less amount of indisposition. The patient feels an extraordinary degree of languor, and has an extremely irritable and variable pulse; the heart and vascular system are greatly excited without any apparently sufficient reason, inasmuch as no organ in especial seems to suffer. Nervous symptoms are, at the same time, manifested; confusion of thought, rambling talk in the night, dry tongue, burning thirst, hot dry skin, trembling of the extremities,—the symptoms, in a word, are those that usher in an attack of common continued fever; but they do not increase in intensity, and after about a fortnight, the patient begins to hack and cough; the nervous symptoms abate, and now it becomes obvious that he is laboring under a serious affection of the chest, which, in the event of the tubercular exudation, which is its element, having been extensive, carries him off in the course of a few weeks in what is called a *galloping consumption.*

Dr. Haller also observed the primary formation of tubercle to take place upon several occasions, along with symptoms of the well-marked intermittent febrile type. But as the qualities of air and soil that give diseases an intermitting character are wanting in this country, we shall not follow the writer here.

With his excellent opportunities of observing incipient tuberculosis, Dr. Haller is enabled from certain signs to perceive the disease before it appears, and when it is only threatened. These signs are mental and corporeal. Those who are about to become the victims of tuberculation of the lung, are either greatly depressed and miserable, or they show a singular irritability and changeableness of disposition. The sleep is generally disturbed by distressing dreams. The subject of observation loses his healthy look and gets thin; sometimes the appetite is increased, sometimes it is greatly diminished; the digestion is imperfect, the bowels are moved more frequently, and the evacuations are larger than usual. Frequently the most remarkable symptoms are referable to disturbance of the circulation; there are symptoms of determination to the head, vertigo, headache; now and then epistaxis, and the individual is subject to change of color constantly; there are symptoms of congestion about the chest, a passing sense of oppression, some cough, perhaps slight hæmoptysis, though this occurs for the most part at a subsequent period; palpitation readily excited, and so obstinate as often to cause suspicions of organic diseases of the heart; indeed, it is only by the use of the stethoscope that the true state of affairs can be discovered.—*Lond. and Edin. Monthly Jour. of Sci.*, Nov. 1841, from *Med. Jahrb. des Œsterreich, Staates*, July, 1841.

